# Effect of Radio Frequency Heating on Yoghurt, II: Microstructure and Texture

**DOI:** 10.3390/foods3020369

**Published:** 2014-06-20

**Authors:** Caroline Siefarth, Thi Bich Thao Tran, Peter Mittermaier, Thomas Pfeiffer, Andrea Buettner

**Affiliations:** 1 Department of Chemistry and Pharmacy, Emil Fischer Centre, Friedrich-Alexander Universität Erlangen-Nürnberg, Schuhstr. 19, Erlangen 91052, Germany; E-Mail: caroline.siefarth@fau.de; 2 Fraunhofer Institute for Process Engineering and Packaging (IVV), Giggenhauser Str. 35, Freising 85354, Germany; E-Mails: thi_bich_thao_tran@cargill.com (T.B.T.T.); peter.mittermaier@ivv.fraunhofer.de (P.M.); thomas.pfeiffer@ivv.fraunhofer.de (T.P.)

**Keywords:** heating, microstructure, radio frequency, cryo-SEM, texture, yoghurt

## Abstract

Radio frequency (RF) heating was applied to stirred yoghurt after culturing in order to enhance the shelf-life and thereby meet industrial demands in countries where the distribution cold chain cannot be implicitly guaranteed. In parallel, a convectional (CV) heating process was also tested. In order to meet consumers’ expectations with regard to texture and sensory properties, the yoghurts were heated to different temperatures (58, 65 and 72 °C). This second part of our feasibility study focused on the changes in microstructure and texture caused by post-fermentative heat treatment. It was shown that there were always microstructural changes with additional heat treatment. Compared to the dense and compact casein network in the stirred reference yoghurt, network contractions and further protein aggregation were observed after heat treatment, while at the same time larger pore geometries were detected. The changes in microstructure as well as other physical and sensorial texture properties (syneresis, hardness, cohesiveness, gumminess, apparent viscosity, G’, G’’, homogeneity) were in good agreement with the temperature and time of the heat treatment (thermal stress). The RF heated products were found to be very similar to the stirred reference yoghurt, showing potential for further industrial development such as novel heating strategies to obtain products with prolonged shelf-life.

## 1. Introduction

Yoghurt is a cultured milk product, typically obtained by fermentation with *Lactobacillus delbrueckii* subsp. *bulgaricus* and *Streptococcus salivarius* subsp. *thermophilus*. These bacterial strains live symbiotically and thus activate each other and promote each other’s growth. Their primary function is to generate lactic acid, amongst others, by fermentation of lactose. In the course of the associated changes in the pH of yoghurt milk, casein micelles are affected, further physico-chemical interactions with other milk components occur, and an acidic milk gel is formed in which the aqueous phase and milk fat are entrapped [1,2].

Depending on the mechanical forces, for example due to stirring or pumping the yoghurt mass following gelation, the casein network can be disrupted, irreversibly leading to stirred yoghurt products with a smooth and viscoelastic texture. According to Walstra [3], the most interesting physical characteristics of either set-style or stirred yoghurt gels include their heterogeneity/homogeneity of structure, fineness, fragility, stiffness (G’), tendency to exhibit syneresis, and gel strength (breaking stress). In any case, the texture of acidic milk gels is an important quality characteristic and the presence of any hard or irregularly formed particles may affect the sensory perception and, consequently, consumer acceptance.

An additional heat treatment after fermentation can further affect the texture of yoghurt [4]. In the literature there is hardly any information about the microstructural and textural changes caused by further heat treatment of yoghurt after fermentation. Chandan & Kilara [5] mentioned a post-ripening heat treatment and claimed that it was possible, together with the use of appropriate stabilizers and a further homogenization process, to redevelop the texture and body of the yoghurt. However, a detailed study comparing such products, for example with and without stabilizer(s), has not yet appeared.

A few patents were published in the late 1970s and 1980s on the production of sterilized yoghurt products (for example, reference [6,7,8,9,10]). Barua & Hampton [6] and Keefer & Murray [7] aimed to produce sterile natural yoghurt having the taste and texture of natural yoghurt. In these studies, and in contrast to the approach described in a patent of Egli & Egli [8], a mix of additives was added in a single step to the product after fermentation, resulting in less complex and less time-consuming processing to obtain sterile yoghurt. Also Amrein & Widmer [9] and, more than ten years later, Güldas & Atamer [10] improved the processing step by adding just a single stabilizer to the yoghurt product after fermentation and before an additional pasteurization or sterilization. The effect of post-fermentative heat treatment without any addition of stabilizers was reported by Bach [11], who applied small temperature changes to yoghurts filled in plastic containers via electromagnetic fields. Nevertheless, besides mentioning a smooth and non-gritty texture, the authors of the above cited patents and publications generally provided no further information about the resulting yoghurt texture or about textural changes caused by the respective additional heat treatment.

In the first part of this two-part study, the applicability of post-fermentative radio frequency (RF) heating and its effect on the shelf-life, pH, color, aroma, and taste of yoghurt were discussed [12]. It was found that living lactic acid bacteria were reduced in number but still present in the RF heated yoghurts, while yeasts and moulds were no longer detected. Also, the aroma and taste profiles were found to be very similar to the reference yoghurt, overall supporting the potential to prolong the yoghurts’ shelf-life.

Based on these observations, this second part of the study investigates the effect of gentle post-fermentative heat treatment on yoghurt microstructure and texture. Following Bach [11], temperatures of 58, 65 and 72 °C, respectively, were applied to a stirred reference yoghurt, either via RF heating or convectional (CV) heating in a convection oven. Microstructural changes were investigated via cryo-scanning electron microscopy (cryo-SEM), and texture changes were measured by texture profile analysis (TPA), rheometry (rotation and oscillation analysis), and measurements on whey separation (syneresis). Textural changes in the yoghurt curd on storage were analyzed over several weeks. Additionally, a trained sensory panel performed sensory texture profile analysis (STPA) during the whole storage period.

## 2. Experimental Section

### 2.1. Yoghurt

Commercially available creamed yoghurt (3.8% fat, 4.4% protein, 5.4% carbohydrates) was purchased from a local supermarket. The yoghurt was filled in 500 mL glass jars, which were closed with a metal screw cap (twist-off, 70 mm). The jars had a diameter of 87 mm and were filled up to about 100 mm. All yoghurts were purchased at the same time and had the same best-before date, which was four weeks after start of the storage period of the present study.

For further investigations, a set-style yoghurt reference (3.8% fat, 3.4% protein, 4.4% carbohydrates), matured in the cup (150 mL), was purchased from a local supermarket and stored with the other yoghurt samples.

### 2.2. Heat Treatment

Prior to heat treatment, the yoghurt samples were pre-tempered to 40 ± 1 °C. The target temperatures of 58, 65 and 72 °C were applied. Immediately after heating, the glass jars were placed in an ice water bath for rapid cooling. For a storage period of five weeks, the samples were placed into a cooling chamber at refrigeration temperatures of 8 ± 1 °C. Samples without any additional heating step were held as references for comparative investigations.

#### 2.2.1. Radio Frequency (RF) Treatment

RF heating of yoghurt was performed in a RF water bath [12]. The 27.12 MHz RF generator was a free-running tube oscillator 16,000 K with 16 kW nominal power and an impedance matching network by the manufacturer Kiefel AG (Freilassing, Bavaria, Germany). The power flow to the products was controlled by automatically adjusting the electrode voltage, which was measured directly at the electrodes. Applied electrode voltages during the yoghurt heating trials were between 2.2 and 2.4 kV, the resulting field intensities in the water bath were between 17.8 and 19.2 kV·m^−1^. By additional control of exposition time, the temperature in the product at the end of RF heating could be determined with narrow tolerance [12].

*Heating procedure*: Two glass jars with tightly closed screw caps standing on a support were immersed in the water bath under atmospheric pressure. The water bath was preheated to a temperature slightly above the process target temperature (+5 K). RF exposition started immediately after immersion and continued for a pre-set duration of time. The electrode voltage and the exposition times had been determined in previous heating experiments according to the respective target temperatures and were 2.3 ± 0.1 kV for 60 s (58 °C), 90 s (65 °C), and 120 s (72 °C), respectively. Samples for further microbiological, structural, textural, and sensory evaluation were exposed to an additional temperature holding time of 60 s and were not subjected to inline-temperature measurements. Further details on the experimental set-up and temperature measurements are described in the first part of this two-part study [12].

#### 2.2.2. Convectional (CV) Treatment

CV heating of yoghurts was performed in a convection oven (Rational white-efficiency SCC 61, Rational AG, Landsberg am Lech, Germany) in steam mode.

*Heating procedure*: Five glass jars with tightly closed screw caps were placed on a fence in the centre of the convection oven. The steam temperature was set to a temperature slightly above the process target temperatures (+5 K). An inline-thermocouple controlled the product temperature with its tip placed in the core of one additional yoghurt jar. Therefore, the lid of the jar was provided with a center hole, and the sample was discarded afterwards. The samples were exposed to CV heating until the process target temperature and an additional temperature holding time of 60 s were reached. For all three temperature regimes, a CV exposition time of *ca.* 60 min was necessary [12].

### 2.3. Structural Analysis via Cryo-Scanning Electron Microscopy (Cryo-SEM)

Directly after heat treatment, about 50 mg samples of each yoghurt and the stirred reference were collected using an ice cream scoop. These samples were flash frozen in liquid nitrogen (−196 °C) and stored at −80 °C to maintain the microstructure of the products. For cryo-preparation, a small piece (about 5 mg) was cut out of the center part of the flash-frozen yoghurts using a cryo-frozen knife. This small fraction was mounted onto a sample carrier using a cryo-glue consisting of agar-agar. Immediately after preparation, the specimen was plunged into liquid nitrogen at atmospheric pressure (−196 °C) and further cooled down to −210 °C. The frozen specimen was transferred under vacuum to a Polaron PP2000T preparation stage (Quorum Technologies Ltd., Lewes, UK) and freeze-fractured at −150 °C with a cold scalpel blade. Subsequently, the fractured specimen was freeze-etched at −80 °C for 15 min and sputter coating was performed at −150 °C with 5 nm of platinum. The platinum-coated specimen was transferred under vacuum into an ABT-55 scanning electron microscope (ISI ABT, Akashi Beam Technology, Tokyo, Japan) using a transportable cryo-stage. On that cryo-stage, the specimen was maintained at a minimum of −130 °C and imaged at an accelerating voltage of 3 kV.

### 2.4. Whey Separation (Syneresis)

Modified by Kessler [13], the whey drip-off volume of intact yoghurt gels was estimated using a drainage test for set-style yoghurts. The test for syneresis was performed for 2 h at 8 ± 1 °C and over the entire storage period (Weeks 0, 2, 4, and 5). The yoghurt jars were placed upside down on a mesh with a mesh size of 1 × 1 mm. The mesh was tightly stretched on a funnel, which was placed on top of a graduated cylinder (100 mL). The whey drip-off volume was recorded in quadruplicate on independent yoghurt jars.

### 2.5. Textural Analysis via Texture Analyzer and Rheometer

Textural analyses were performed on the intact and stirred yoghurt gels. The texture was analyzed throughout the entire storage period of five weeks in the following intervals: Weeks 0, 2, 4, and 5. At Week 0, a set-style reference was additionally investigated. For all texture analyses, the samples were kept in the refrigerator (8 ± 1 °C) until analysis. The texture of each sample was analyzed in quadruplicate on independent yoghurt jars.

#### 2.5.1. Intact Yoghurt Gels

**Figure 1:**
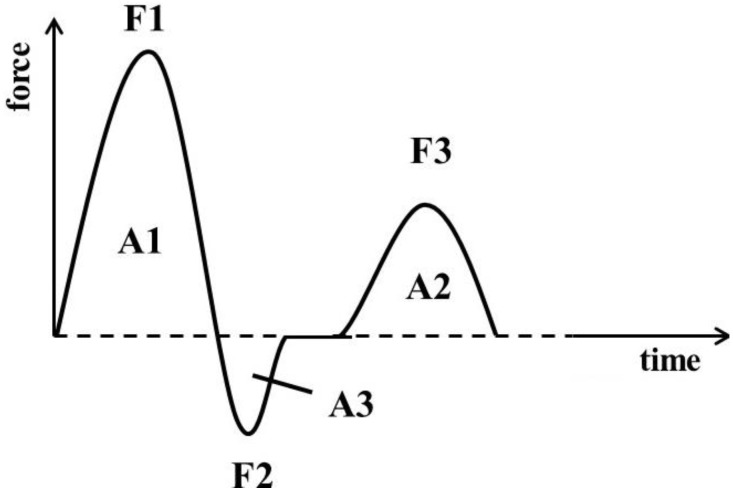
Force-time curve of texture profile analysis (TPA) with F—force and A—area under curve, modified from Rosenthal [16].

The texture profiles of the intact yoghurt gels were analyzed directly in the yoghurt jars to avoid any damage or modifications of structure. Texture profile analysis (TPA) was performed with a TA.XTplus Texture Analyzer (Stable Microsystems Ltd., Surrey, UK) equipped with a 50 kg load cell. Two sequential compression events were performed with an aluminum cylinder probe (25 mm, P/25, Stable Microsystems) with a diameter that was about a third of the jar diameter to minimize side-wall effects [14]. The TPA settings modified from Kaur *et al.* [15] were 12 mm penetration at 0.5 mm·s^−1^ during compression as well as pre-test and post-test speed. During the measurements, the yoghurt jars were held manually on the base plate. The texture profile was analyzed via Exponent software (Stable Microsystems). Typical force-time curves, imitating the conditions in mouth after two subsequent product compressions, were obtained as shown in Figure 1.

Data on forces (F) and areas under curve (A) of the force-time curves were used to calculate the following TPA parameters as described previously by Bourne [14]: *hardness* (HD, (g)), as maximum force of first compression cycle (=F1); *cohesiveness* (CO), as ratio of the positive force areas under first and second compression and thus, parameter representing the strength of internal bonds or so called inner gel strength (=A2/A1); *adhesiveness* (AD, (g·s)), as negative force area under the first bite (=A3) and thus, parameter representing the work to pull the cylinder probe out of the sample which is correlated with removing a sample from the mouth during eating that adheres to tongue and mucosal tissues [15]; and *gumminess* (GU, (g)), as product of HD and CO and thus, the energy required to treat the food for being ready to be swallowed.

#### 2.5.2. Stirred Yoghurt Gels

The yoghurt gels were stirred manually by use of a spoon, which was rotated very quickly. The procedure was performed until the yoghurts appeared to be very homogenous, with a number of rotations from 20 to 25.

The stirred yoghurt gels were analyzed with a Bohlin CVO50 rheometer (Malvern Instruments Ltd., Worcestershire, UK) using a parallel plate geometry (PP40) with a gap size of 1 mm. Both, rotational measurements as well as oscillatory measurements were carried out to describe the overall flow properties of the viscoelastic samples [17]. The following tests were performed according to Vercet *et al.* [18] and Afonso & Maia [19]: (1) *Flow*. Up-and-down flow curves were performed at shear rates from 10 to 290 s^−1^. Overall, 26 data points were obtained with a delay time of 5 s. (2) *Viscosity*. Apparent viscosity (η) was determined within the linear viscoelastic section of the flow curve for 2 min. The difference in initial and final apparent viscosity was calculated using the following formula adopted from Vercet *et al.* [18]: % broken structure = [(initial viscosity − final viscosity)/initial viscosity] × 100. (3) *Oscillation*. An amplitude sweep was performed at a frequency of 5 Hz and strain amplitude from 0.1% to 100% to determine the viscoelastic region of the yoghurt samples. Further oscillation tests for the determination of storage modulus (G’, representing the elastic behavior of the yoghurt sample) and loss modulus (G’’, representing the viscous behavior of the yoghurt sample) were performed at 5 Hz and 2% strain, which was found to be inside the linear viscoelastic range.

### 2.6. Sensory Texture Profile Analysis (STPA) and Hedonic Rating

Stirred yoghurt samples (20 mL) were filled into sensory glass beakers (140 mL, J. Weck GmbH u. Co. KG, Wehr, Germany) and closed with a lid. STPA were performed in a sensory panel room at 21 ± 1 °C. Trained panelists (*n* = 13, male/female, 23 to 45 years) from the Friedrich-Alexander University of Erlangen-Nürnberg (Erlangen, Germany) and Fraunhofer IVV (Freising, Germany) participated in the STPA sessions and exhibited no known illness at the time of examination. The panelists were asked to score the textural properties on a visual analogue scale (10 cm). Prior to this study, the assessors were recruited in a training session on the evaluation of the following texture attributes adapted from Clark *et al.* [20]: *creaminess*, from watery (0) to creamy (10); *stickiness*, from sticky and thus, adherent to the mouth (0) to soft (10); *homogeneity* (mouthfeel), from grainy (0) to smooth/homogenous (10); and *visual homogeneity* (“v”), from grainy (0) to smooth/homogenous (10).

The order of presentation of the different samples was randomized and no information on the purpose of the experiment or the composition of the samples was given to the panelists. STPA were performed in the following intervals: Weeks 0, 2, and 4. Since the best-before date of the reference yoghurt expired after four weeks of storage, no further sensory testing was performed afterwards. Additionally, a set-style yoghurt reference was evaluated by the panelists at Week 0. The results for each texture attribute and sample were averaged and plotted in a box-plot diagram.

For the hedonic rating, the panelists were asked to rate the pleasantness of the differently treated yoghurt samples on a scale from 1 (dislike extremely) through 5 (neither like nor dislike) to 9 (like extremely).

### 2.7. Statistical Analysis

Statistical analyses were performed using the software OriginPro 9G (OriginLab Co., Northampton, MA, USA) and Statistica 10 (StatSoft Europe GmbH, Hamburg, Germany), respectively. One-way analysis of variances (ANOVA) and Fisher LSD *post-hoc* testing were carried out to elaborate differences between the differently treated yoghurts and over storage time (repeated measures ANOVA) for all groups of data. The level of statistical significance was set at 5%.

## 3. Results and Discussion

Gentle radio frequency (RF) heating was applied to stirred yoghurt, a matrix that represents an acidic casein gel with a very sensitive structure. In the following section, the observed microstructural and textural effects associated with this post-fermentative heat treatment are discussed and compared to the effects of the respective convectional (CV) heating process.

Thereby, it has to be noted that it was decided to abstain from the RF 72 °C heating in the heating series. As already described in the first part of this study [12], it was not possible to establish a stable heating regime for RF 72 °C products since in some cases a significant overheating followed by strong contraction of the yoghurt curd and whey separation was observed. The few 72 °C samples of the heating experiments were kept, and textural analyses were performed at Week 0. Further textural analyses, as well as microstructural analyses and sensory texture profile analyses (STPA) were only performed on yoghurts heated to 58 °C and 65 °C.

### 3.1. Microstructural Analysis via Cryo-SEM

**Figure 2:**
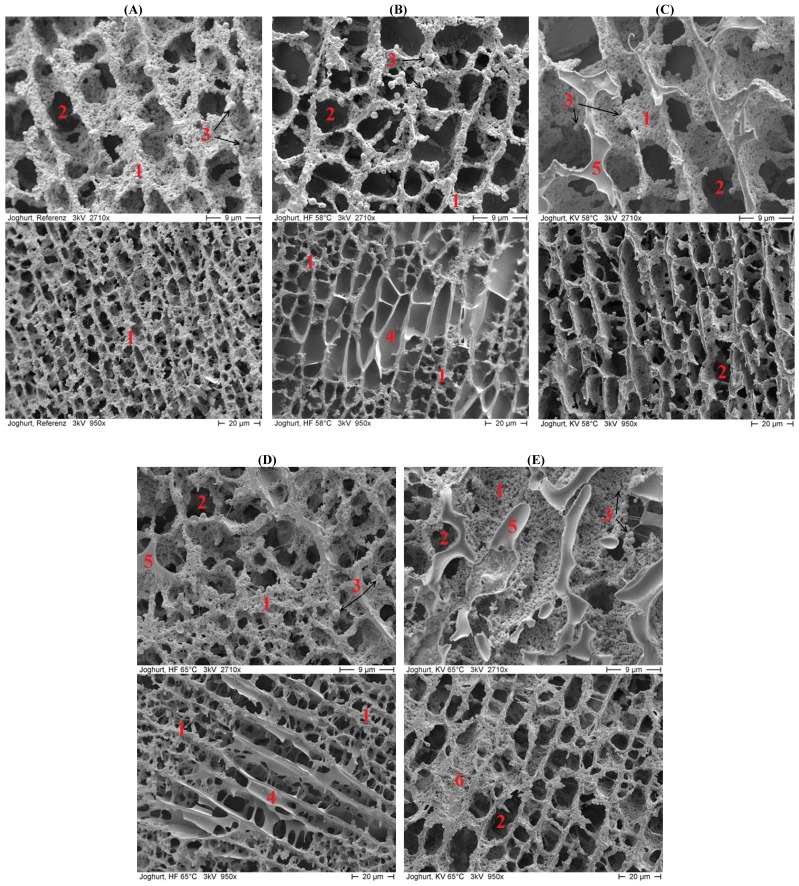
Microstructure of heated yoghurt gels (obtained by cryo-scanning electron microscopy (SEM)) at different magnification: (**A**) Reference; (**B**) radio frequency heating (RF) 58 °C; (**C**) convectional heating (CV) 58 °C; (**D**) RF 65 °C; (**E**) CV 65 °C; with **1**—network of micellar caseins linked together in clusters, chains, and strands; **2**—pores originally confining whey; **3**—fat globules; **4**—unknown formations within the network; **5**—frozen water. Microstructural observations are based on similar observations reported by other authors [22,23].

Cryo-SEM is a very valuable technique in dairy research. The latter fact is related to the rapid freezing and sample handling and thus to the formation of vitreous ice with an amorphous, non-crystalline structure which prevents the occurrence of any deformation or distortion of the microstructure [21]. The results of cryo-SEM are shown in Figure 2 at two different magnifications.

As expected from literature surveys, cryo-SEM was able to provide detailed information about changes to the yoghurt’s microstructure caused by post-fermentative heat treatment (Figure 2). The gel structure of yoghurt is a three-dimensional network of micellar caseins, which are linked together in clusters, chains, and strands. According to Lee & Lucey [24], acidic gels made from heated milk are reported to be highly branched and thus possess a fine structure as can be seen in Figure 2A in the cryo-SEM image of the reference yoghurt. The network is very homogeneous, showing a well-defined and compact structure of micellar caseins (**1**) with intact pores inside the casein network originally confining the milk serum phase (whey) (**2**), as reported before by Hassan *et al.* [22] and Sandoval-Castilla *et al.* [23]. Fat globules (**3**) are also entrapped within the protein network. Due to homogenization processes of the yoghurt milk, their size was reduced to about 2 μm [25].

As can be seen in Figure 2B–E, the casein network changed due to post-fermentative heat-treatment. In the case of RF heating (Figure 2B,D), the micellar casein strands appear to undergo a contraction process, resulting in gel microstructures with larger interstitial spaces (**2**). Fat globules (**3**), on the other hand, are less entrapped in the network even if their size remains stable (~2 µm). In addition, some unknown formations (**4**) presumably filled with milk serum phase can be observed after RF heating, which are segregated from the protein network and remain intact after mild heat treatment at 58 °C (Figure 2B, **4**) but are disrupted after treatment at 65 °C (Figure 2D, **4**). A closer look at the cryo-SEM images at lower magnification shows that these unknown formations are partially distributed across the casein network; however, overall, the network of micellar caseins still appears uniform and very similar to the reference but with larger interstitial spaces. After CV heating, the visual appearance of the casein network is very dense with even larger interstitial spaces (Figure 2C,E, **2**). Moreover, large regions of frozen water are seen on the surface layer (Figure 2C,E, **5**) as observed by Hassan *et al.* [22] who also investigated the structural properties of yoghurts and other dairy products using cryo-SEM. In contrast, small amounts of frozen water were only observed for RF heating at 65 °C (Figure 2D, **5**).

In previous studies, there were hardly any reports of physico-chemical interactions that might occur due to post-fermentative heating, thereby leading to an open structure with large(r) pores. With any network contraction, not only the milk serum in the networks’ pores but also the serum entrapped in the casein network in the form of capillary water (between micellar casein chains and strands, ~10%–30% of total milk serum) and hydration water (~40% of total milk serum) [26] might be affected and emanate. Such an effect could lead to increased amounts of free serum phase, the formation of unknown conglomerates within the network after RF heating (Figure 2B,D, **4**), and/or large amounts of frozen water after CV heating (Figure 2C,E, **5**). Indeed, Töpel [26] reported that the amount of capillary-bound water was strongly related to the network’s microstructure, with decreasing amounts of enclosed water with opening of the structure, thereby forming a coarse-meshed network (Figure 2B–E). Another aspect that has to be considered is (further) protein aggregation due to an additional heat treatment, as can be suspected to have occurred in Figure 2E, **6**. Certainly, on the macro-scale, the formation of white flakes (lumps) was observed by direct visual inspection in the stirred yoghurts both after RF and CV heating; these flakes increased in number and size with increasing temperature and duration of heat treatment. It is known from the manufacturing process for stirred yoghurts that textural defects, known as lumpiness or graininess, can occur [27]. Töpel [26] reported flocculation in heated milk products in the context of surface modifications of micellar caseins due to low pH and denatured whey proteins at the caseins’ surface. Thus, dense aggregates as seen in cryo-SEM image Figure 2E, **6** may reveal the presence of white flakes, which are reported in the range of 1 to 5 mm [28]. Such textural defects are known to be reversible and can be removed by stirring, low-pressure homogenization or pumping the yoghurt mass through special devices such as meshes, filters, smoothening valves or colloid mills [27,29].

### 3.2. Whey Separation (Syneresis)

In a further part of the study, whey drainage volumes were investigated in order to observe changes within the yoghurt curds due to the post-fermentative heat treatment. All products were analyzed before stirring and thus still had their intact gel network. Data on whey separation over the whole storage period is shown in Table 1.

Whey separation was observed in all the heated yoghurts (Table 1). In general, whey separation is a commonly detected long-term storage effect of yoghurt products, since yoghurt is a dairy product with a very high percentage of water content [24]. Nevertheless, in our case the reference yoghurt showed no syneresis over the entire storage period. Accordingly, the observed syneresis effects were directly linked to the heat treatments that were performed.

As reported in the first part of our study [12], for some cases of RF 72 °C heated products a significant overheating was observed during manufacturing followed by strong contraction of the yoghurt curd and whey separation. In detail, the separated whey volume after 72 °C RF heating was greater than 50 mL and thus about 1/10 of the original yoghurt volume. With around 20 mL, the 72 °C CV samples also showed a significantly higher syneresis volume than yoghurts heated to lower temperatures at Week 0. For CV heating to 65 °C, significant differences in whey drainage volumes were observed compared to RF heating and CV heating to 58 °C (Weeks 0, 4) (Table 1). After a storage period of two weeks, significant differences in whey drainage volumes were found between the RF and CV products in general.

Via cryo-SEM, different pore radii within the casein network became visible, as well as some unknown formations presumably filled with whey (4) and frozen water (5) (Figure 2). This information about the yoghurts’ microstructure explains the increasing whey drainage volumes with increasing thermal stress. As mentioned in Section 3.1, a decrease in capillary-bound water or even hydration water could have been responsible for an increase in free serum phase and thereby syneresis volumes [26].

On storage, the whey drainage volumes increased for the RF heated products and the CV heated samples showed the same tendency (*F* = 2.63 (n.s.), 13.99 (sig.), 11.87 (sig.), 3.57 (n.s.); *p* < 0.05 (RF 58 °C, RF 65 °C, CV 58 °C and CV 65 °C)). These results are in line with the findings of other researchers on the syneresis behavior of yoghurt products on long-term storage [30]. Presumably, structural rearrangements occur within the gel networks during storage, causing a breakage of casein strands and fusion processes of casein particles [24].

### 3.3. Textural Analysis via Instrumental Assessment

#### 3.3.1. TPA on Intact Yoghurt Gels

The textures of differently treated intact yoghurts gels were evaluated using a texture analyzer by means of the following attributes: hardness (HD), cohesiveness (CO), adhesiveness (AD) and gumminess (GU), respectively. The results for different storage periods are shown in Table 2 (Week 0) and Table 3 (Weeks 2, 4, 5).

**Table 1:** Whey separation (mL) on second heat treatment (RF, CV) of yoghurt to different temperatures including results of analysis of variance (ANOVA) (one-way).

Whey Separation (mL)	Reference Yoghurt	RF-Treated Yoghurts			CV-Treated Yoghurts			ANOVA Results	
		58 °C	65 °C	72 °C	58 °C	65 °C	72 °C	*F* Value	*p* Value
Week 0	ND (0.0 ± 0.0)	9.5 ± 1.4a,b	6.7 ± 0.7a	56.2 ± 10.0d	7.6 ± 2.0a	13.7 ± 1.0b	19.3 ± 4.2c	74.88	1.59 × 10^−10^
Week 2	ND (0.0 ± 0.0)	9.2 ± 0.2a	8.5 ± 2.7a ^1^	-	18.5 ± 1.5b	20.0 ± 8.1b ^1^	-	6.75	7.57 × 10^−3^
Week 4	ND (0.0 ± 0.0)	9.6 ± 7.6a ^1^	12.8 ± 2.9a	-	14.0 ± 1.5a	22.9 ± 3.5b	-	6.45	7.56 × 10^−3^
Week 5	ND (0.0 ± 0.0)	16.1 ± 2.0b	15.4 ± 1.5a,b	-	11.2 ± 4.3a ^1^	15.1 ± 1.0a,b	-	2.92	8.16 × 10^−2^

^1^ Coefficient of variation >25%. Intensity values with different letters indicate significant differences between products (*p* < 0.05, Fisher LSD *post-hoc*).

**Table 2:** Rheological parameters of intact yoghurt gels and results of ANOVA (one-way): week 0.

Texture Profile Analysis (TPA)	Reference Yoghurt	RF-Treated Yoghurts			CV-Treated Yoghurts			Set Reference Yoghurt	ANOVA Results	
		58 °C	65 °C	72 °C	58 °C	65 °C	72 °C		*F* Value	*p* Value
Hardness HD (g)	32.961 ± 2.660a	57.271 ± 1.577b	99.066 ± 12.430d	86.605 ± 6.814c	112.142 ± 4.848e	107.038 ± 3.689de	142.693 ± 11.755f	160.936 ± 5.282g	102.38	2.22 × 10^−16^
Cohesiveness CO	0.617 ± 0.016a	0.543 ± 0.040b	0.487 ± 0.007c	0.570 ± 0.011b	0.466 ± 0.019c	0.489 ± 0.015c	0.455 ± 0.025c	0.390 ± 0.031d	33.33	7.28 × 10^−11^
Adhesiveness AD (g·s)	73.000 ± 12.268bc	126.000 ± 11.591d	115.522 ± 21.873d	0.003 ± 0.005a^1^	81.566 ± 18.221bc	49.537 ± 10.743b	54.465 ± 38.268b^1^	101.750 ± 20.092cd	13.03	8.25 × 10^−7^
Gumminess GU (g)	20.293 ± 1.176a	33.064 ± 1.295b	48.215 ± 5.769c	49.331 ± 3.489c	51.356 ± 0.582c	51.643 ± 2.293c	64.648 ± 2.525d	62.818 ± 5.263d	60.36	1.08 × 10^−13^

^1^ Coefficient of variation >25%. Intensity values with different letters indicate significant differences between products (*p* < 0.05, Fisher LSD *post-hoc*).

**Table 3:** Rheological parameters of intact yoghurt gels and results of ANOVA (one-way): Week 2, 4, 5; with hardness (HD), cohesiveness (CO), adhesiveness (AD) and gumminess (GU).

Texture Profile Analysis (TPA)		Reference Yoghurt	RF-Treated Yoghurts		CV-Treated Yoghurts		ANOVA Results	
			58 °C	65 °C	58 °C	65 °C	*F* Value	*p* Value
Week 2	HD (g)	33.379 ± 0.962a	57.646 ± 3.626b	96.335 ± 13.917c	90.643 ± 2.195c	114.678 ± 13.599d	40.19	7.57 × 10^−8^
	CO	0.596 ± 0.010a	0.532 ± 0.015b	0.487 ± 0.011c	0.495 ± 0.011c	0.480 ± 0.022c	38.90	9.44 × 10^−8^
	AD (g·s)	89.750 ± 20.584a	113.000 ± 14.612b	89.750 ± 19.434a	65.000 ± 4.583a	83.750 ± 21.487a ^1^	2.95	5.54 × 10^−2^
	GU (g)	19.771 ± 0.357a	30.621 ± 1.274b	46.818 ± 6.050c	44.847 ± 1.453c	58.031 ± 1.445d	80.68	5.87 × 10^−10^
Week 4	HD (g)	32.037 ± 0.723a	81.367 ± 2.685b	104.357 ± 17.762c	104.933 ± 7.409c	121.986 ± 11.941c	35.28	1.82 × 10^−7^
	CO	0.602 ± 0.014a	0.511 ± 0.016b	0.483 ± 0.015c	0.483 ± 0.012c	0.473 ± 0.014c	46.50	2.80 × 10^−8^
	AD (g·s)	69.000 ± 11.247a	120.000 ± 9.721b	77.750 ± 46.885a ^1^	82.500 ± 11.715ab	69.250 ± 15.610a	2.38	9.78 × 10^−2^
	GU (g)	19.265 ± 0.053a	41.598 ± 1.457b	50.247 ± 7.780c	50.596 ± 2.700c	57.523 ± 4.429c	37.19	1.28 × 10^−7^
Week 5	HD (g)	42.390 ± 2.285a	80.797 ± 0.986b	98.642 ± 16.684c	101.107 ± 8.037c	135.789 ± 2.174d	51.64	1.36 × 10^−8^
	CO	0.545 ± 0.004a	0.503 ± 0.012bc	0.509 ± 0.047ab	0.486 ± 0.014bc	0.466 ± 0.013c	4.95	9.53 × 10^−3^
	AD (g·s)	103.000 ± 6.595bc	104.250 ± 23.626c	62.500 ± 9.206a	65.250 ± 19.227a ^1^	77.250 ± 6.942ab	5.50	6.28 × 10^−3^
	GU (g)	23.078 ± 1.099a	40.596 ± 0.726b	49.489 ± 4.825c	49.018 ± 2.725c	63.196 ± 0.840d	104.99	8.88 × 10^−11^

^1^ Coefficient of variation >25%. Intensity values with different letters indicate significant differences between products (*p* < 0.05, Fisher LSD *post-hoc*).

Directly after manufacturing, there was a significant difference between the reference and all the heat-treated yoghurt samples with respect to the TPA attributes HD, CO and GU (Table 2). For these attributes, the RF 58 °C yoghurt was closest to the reference product and differed significantly from the RF 65 °C sample and the yoghurts treated via CV heating.

**Figure 3:**
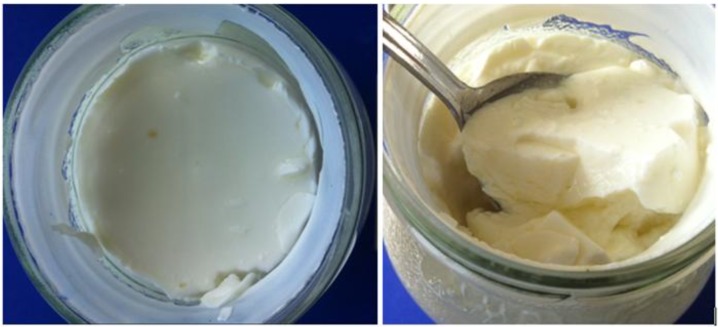
Visual appearance of intact yoghurt gels after post-fermentative heat treatment and before stirring (here: CV 65 °C).

In detail, the maximum force of first compression (HD) increased with increasing temperature of the post-fermentative heat treatment and with heating time (Reference < RF 58 °C < RF 65 °C < CV 58 °C and 65 °C < CV 72 °C) (Table 2). The highest HD value was obtained for the set-style reference yoghurt. Thus, it appears that an additional heat treatment alters the texture of the yoghurt from stirred towards set-style, an observation that can also be confirmed by the visual appearance of the intact yoghurt samples within the glass jar before stirring as seen in Figure 3 (here: CV 65 °C). The top view of the glass jar shows the yoghurt curd to be contracted, firm, and textured, attributes that are typical for set-style yoghurt. The only “outlier” in Table 2 is the RF 72 °C product. As described above and according to the first part of this study [12], it was not possible to establish a stable heating regime for this product temperature. In some cases, significant overheating was observed followed by strong contraction of the yoghurt curd and whey separation. Hence, unknown (structural) changes within the product are the most likely reason for the statistical deviation.

The inner gel strength, represented by CO, decreased with increasing temperature and duration of heat treatment (Table 2). The set-style reference showed the lowest inner gel strength and differed significantly from the other samples. Thus, the inner gel strength of the RF and CV heated yoghurts also changed more and more towards a set-style yoghurt. The parameter GU, representing the energy required to get the food ready for swallowing again, increased with increasing temperature and heating time. Thus, increasing HD values for the acidic casein gels and decreasing inner gel strengths should impair, to a certain degree, the swallowing of the yoghurt. The parameter AD, representing the effort required to remove a sample from the mouth due to adherence, shows less aligned results in Table 2 for Week 0: with lowest values for the CV heated products, highest values for the RF heated products, and the values of the stirred and set-style references lying in between.

It can thus be concluded that the syneresis volumes increased and the yoghurts’ texture contracted with increasing thermal stress of post-fermentative heat treatment, with a rise in HD and GU values and a decrease in the strength of internal bonds (CO). Roefs *et al.* [31] reported that most textural parameters for the characterization of casein gels depend on the number of bonds between the micellar caseins and thus the three-dimensional distribution of the network’s chains and strands. These considerations of Roefs *et al.* [31] are in good agreement with the findings of the present study on CO values, and are further supported by the cryo-SEM images, e.g., with respect to the density of casein chains and strands or different pore radii).

Table 3 summarizes the data obtained on monitoring the textural changes of the intact yoghurt gels during storage. Once again, the reference yoghurt shows significant differences to the samples that were additionally heat-treated. The RF 58 °C product was most similar to the reference with respect to the textural attributes HD, CO and GU. With temperature and time of heat treatment, these three TPA parameters increased or decreased in the same manner as observed for the Week 0 samples.

Related to the alteration of the yoghurt gels, the HD and GU values of most samples significantly increased in storage while the CO values slightly decreased (HD: *F* = 19.94 (sig.), 100.41 (sig.), 0.12 (n.s.), 16.34 (sig.), 4.41 (sig.); CO: *F* = 32.78 (sig.), 2.34 (n.s.), 0.54 (n.s.), 2.98 (n.s.), 1.33 (n.s.); and GU: *F* = 11.66 (sig.), 62.95 (sig.), 0.16 (n.s.), 8.69 (sig.), 12.57 (sig.); *p* < 0.05 (Reference, RF 58 °C, RF 65 °C, CV 58 °C and CV 65 °C)). As described above for the data for Week 0, the data for the TPA parameter AD during storage did not reveal any specific trend towards a steady increase or decrease. Hence, it can be deduced that in storage there is again a further rise in the HD and GU values and a decrease in the strength of internal bonds (CO). These data on the textural changes during storage are in good agreement with previous findings reported in the literature. According to Lucey [27] and Lee & Lucey [24], shrinkage of the yoghurt network was observed during storage, accompanied by altered related properties (e.g., increasing yoghurt hardness).

#### 3.3.2. Texture Analysis on Stirred Yoghurt Gels

**Figure 4:**
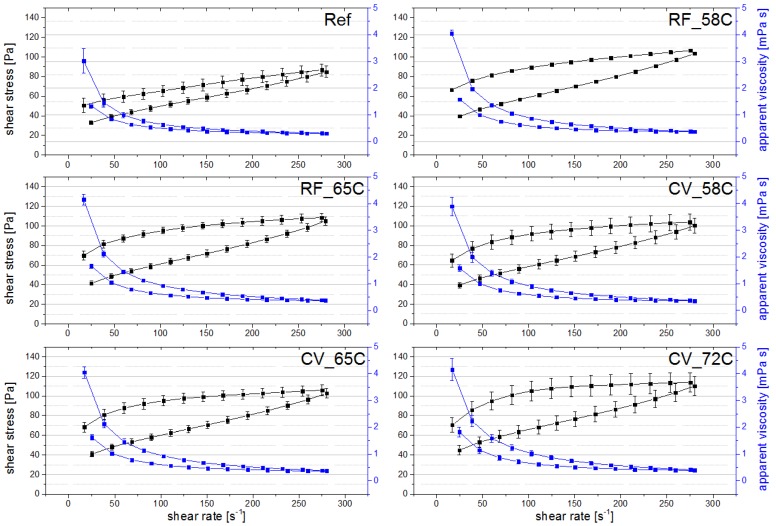
Flow and viscosity curves of yoghurt reference (Ref), RF and CV heated yoghurts to different temperatures (58 °C, 65 °C, 72 °C): shear stress (black) and apparent viscosity (blue).

Rotational measurements as well as oscillatory measurements were carried out by application of rheometry to measure the overall viscoelastic properties of the differently treated yoghurts after stirring. For rotation, up-and-down flow curves were investigated. A shear rate of 112 s^−1^ was found to be within the linear viscoelastic section of the products’ different flow curves (Figure 4). Similar shear rates have been described before and these have been reported to be within the linear viscoelastic region of stirred yoghurts and can thus be used to determine their apparent viscosities [18,32]. Characteristic of yoghurt, the flow, hysteresis, and viscosity curves in Figure 4 indicate a non-Newtonian, shear thinning, thixotropic behavior of all samples. For the thixotropic behavior, the hysteresis areas in Figure 4 were observed to increase with increasing thermal stress.

Results on apparent viscosities are shown in Table 4 (Week 0) and Table 5 (Weeks 2, 4, 5). In accordance with previous reports, all yoghurt samples in Table 4 showed a decrease from initial to final apparent viscosity and thus a time-dependent behavior (e.g., reference [18]). The initial and final apparent viscosity and percental broken structure of the yoghurts significantly increased with increasing temperature regime and time of post-fermentative heat treatment. According to the observations described in Section 3.3.1 about the texture properties of intact yoghurt gels, the reference sample showed significantly different results compared to the heat-treated yoghurts, with the lowest initial and final apparent viscosities, the lowest percental broken structure, and thus the highest gel strength. The stirred RF 58 °C product was again most similar to the reference with respect to its textural properties. The set-style reference showed a low initial and final viscosity but, like the yoghurts heated to 72 °C, a high percentage of broken structure and thus comparably low gel strength.

Table 5 reveals similar findings on apparent viscosity and percental broken structure over the entire storage period compared to week 0 (Table 4). The reference was significantly different compared to the heat-treated yoghurts, with the stirred RF 58 °C yoghurt being closest to the stirred reference. With increasing thermal stress, all the values increased significantly.

On storage, the apparent viscosities and percental broken structures significantly increased for most of the yoghurts (apparent viscosity (initial): *F* = 10.34 (sig.), 19.34 (sig.), 21.54 (sig.), 32.02 (sig.), 1.88 (n.s.); apparent viscosity (final): *F* = 4.36 (sig.), 29.96 (sig.), 8.37 (sig.), 17.87 (sig.), 2.56 (n.s.); and % broken structure: *F* = 4.76 (sig.), 5.56 (sig.), 8.31 (sig.), 12.24 (sig.), 7.97 (sig.); *p* < 0.05 (Reference, RF 58 °C, RF 65 °C, CV 58 °C and CV 65 °C)). These results agree quite well with the TPA findings described in Section 3.3.1, where there was an increase in gel firmness on storage and at the same time a slight decrease in the inner gel strength of the yoghurts.

**Table 4:** Rheological parameters of stirred yoghurt gels and results of ANOVA (one-way): Week 0.

Rotation and Oscillation Analysis		Reference Yoghurt	RF-Treated Yoghurts			CV-Treated Yoghurts			Set Reference Yoghurt	ANOVA Results	
			58 °C	65 °C	72 °C	58 °C	65 °C	72 °C		*F* Value	*p* Value
Apparent viscosity (Pa·s)	Initial	0.852 ± 0.014a	1.079 ± 0.039c	1.141 ± 0.043cd	1.317 ± 0.090e	1.198 ± 0.038d	1.318 ± 0.005e	1.457 ± 0.042f	0.971 ± 0.065b	58.57	1.51 × 10^−13^
	Final	0.555 ± 0.021b	0.621 ± 0.017c	0.632 ± 0.021c	0.449 ± 0.057a	0.653 ± 0.018c	0.698 ± 0.003d	0.737 ± 0.025d	0.453 ± 0.025a	55.78	2.61 × 10^−13^
	% Broken structure	34.875 ± 1.560a	42.464 ± 1.628b	44.588 ± 0.665bc	65.609 ± 5.440f	45.444 ± 0.730bc	47.028 ± 0.317cd	49.434 ± 0.643d	53.273 ± 0.712e	52.52	5.10 × 10^−13^
Oscillatory test (Pa)	G’	385.128 ± 141.595a ^1^	400.585 ± 60.124a	561.600 ± 60.186a	1401.226 ± 389.870c ^1^	591.024 ± 59.334a	576.934 ± 99.024a	868.278 ± 12.132b	644.001 ± 61.057ab	13.23	7.19 × 10^−7^
	G’’	144.374 ± 51.547 a ^1^	124.086 ± 16.562a	173.494 ± 13.801a	436.935 ± 112.452c ^1^	178.469 ± 16.495a	175.900 ± 29.154a	268.420 ± 6.590b	201.644 ± 18.500ab	13.96	4.40 × 10^−7^

^1^ Coefficient of variation >25%. Intensity values with different letters indicate significant differences between products (*p* < 0.05, Fisher LSD *post-hoc*).

**Table 5:** Rheological parameters of stirred yoghurt gels and results of ANOVA (one-way): Weeks 2, 4, 5.

Rotation and Oscillation Analysis			Reference Yoghurt	RF-Treated Yoghurts		CV-Treated Yoghurts		ANOVA Results	
				58 °C	65 °C	58 °C	65 °C	*F* Value	*p* Value
Week 2	Apparent viscosity (mPa·s)	Initial	0.807 ± 0.043a	1.180 ± 0.034b	1.291 ± 0.035b	1.472 ± 0.062c	1.427 ± 0.137c	42.23	5.41 × 10^−^^8^
		Final	0.523 ± 0.024a	0.615 ± 0.010b	0.657 ± 0.033b	0.723 ± 0.027c	0.713 ± 0.055c	19.18	9.19 × 10^−^^6^
		% Broken structure	35.139 ± 0.813a	47.792 ± 1.568b	49.123 ± 1.389bc	50.816 ±1.676c	50.032 ± 1.083bc	69.79	1.64 × 10^−^^9^
	Oscillatory test (Pa)	G’	177.643 ± 22.163a	464.479 ± 264.951b ^1^	829.497 ± 144.263c	671.827 ± 37.555bc	614.750 ± 168.148bc ^1^	7.55	1.53 × 10^−^^3^
		G’’	58.985 ± 6.280a	178.843 ± 11.699b	252.599 ± 52.660c	201.149 ± 10.039bc	189.612 ± 46.672b	14.72	4.47 × 10^−^^5^
Week 4	Apparent viscosity (mPa·s)	Initial	0.909 ± 0.005a	1.304 ± 0.038b	1.369 ± 0.046c	1.389 ± 0.023c	1.452 ± 0.049d	120.27	3.32 × 10^−^^11^
		Final	0.568 ± 0.004a	0.711 ± 0.009bc	0.705 ± 0.005b	0.726 ± 0.011c	0.763 ± 0.021d	129.52	1.94 × 10^−^^11^
		% Broken structure	37.541 ± 0.463a	45.474 ± 1.531b	48.499 ± 1.218c	47.719 ± 0.255c	47.441 ± 0.717c	65.69	2.52 × 10^−^^9^
	Oscillatory test (Pa)	G’	311.313 ± 121.421a ^1^	461.110 ± 89.371ab	696.739 ± 6.195c	652.366 ± 68.599bc	526.338 ± 187.800bc ^1^	5.70	5.39 × 10^−^^3^
		G’’	95.718 ± 33.897a ^1^	142.018 ± 23.555ab	213.894 ± 2.990c	194.065 ± 19.226bc	162.922 ± 52.584bc ^1^	6.59	2.87 × 10^−^^3^
Week 5	Apparent viscosity (mPa·s)	Initial	0.922 ± 0.031a	1.327 ± 0.059b	1.332 ± 0.022b	1.391 ± 0.035bc	1.428 ± 0.045c	80.28	6.08 × 10^−^^10^
		Final	0.580 ± 0.023a	0.712 ± 0.030b	0.706 ± 0.004b	0.731 ± 0.003b	0.736 ± 0.014b	38.04	1.10 × 10^−^^7^
		% Broken structure	37.088 ± 0.963a	46.318 ± 1.656b	46.966 ± 0.778bc	47.415 ± 0.987bc	48.440 ± 0.678c	56.19	7.54 × 10^−^^9^
	oscillatory test (Pa)	G’	173.049± 1.544a	564.869 ± 151.042bc ^1^	534.679 ± 163.877b ^1^	723.439 ± 31.596cd	830.820 ± 27.264d	18.41	1.18 × 10^−^^5^
		G’’	59.458± 2.488a	169.716 ± 41.109bc	164.178 ± 45.164b ^1^	213.616 ± 8.514cd	245.669 ± 8.373d	19.40	8.58 × 10^−^^6^

^1^ Coefficient of variation >25%. Intensity values with different letters indicate significant differences between products (*p* < 0.05, Fisher LSD post-hoc).

As a decrease from initial to final apparent viscosity was reported for all yoghurts, the storage and loss moduli (G’, G’’) were also investigated in the present study and are shown in Table 4 and Table 5. According to Vercet *et al.* [18], a strain amplitude of 2% was found to be within the linear viscoelastic region of the yoghurt samples. With the exception of the 72 °C samples, the storage and loss moduli of the samples were not significantly different directly after manufacturing (Table 4). Accordingly, their viscoelastic properties were quite similar directly after heat treatment. Thereby, the loss tangent (=G’’/G’) was <1.0, as is typical for (yoghurt) gels; thus all yoghurts behaved more like a solid with the storage modulus G’ showing higher values compared to G’’ over the entire linear viscoelastic region [33].

On storage, significant differences in the viscoelastic parameters developed in relation to the applied temperature and time of post-fermentative heat treatment. Again, the stirred reference was significantly different from all the heat-treated samples and RF 58 °C yoghurts were closest to the reference, with the sample of Week 5 being the only exception. More specifically, the lowest G’ and G’’ values were observed for the stirred reference which is in accordance with previous findings of other groups [34,35]. Smith & Hui [36] reported that low G’ and G’’ values were typical for very fine and homogenous yoghurt networks. This in turn correlates with the findings obtained from our cryo-SEM investigations. Lucey *et al.* [37] stated that cross-linking, presumably by heat-denatured whey proteins, could be responsible for increased rigidity and G’ of yoghurt networks.

Regarding changes in the individual yoghurt samples, it was observed that the storage and loss moduli G’ and G’’ of the CV treated samples (slightly) increased with storage time. A significant increase was only found for the CV 65 °C sample: G’, F = 4.16 (sig.); G’’, F = 3.86 (sig.); p < 0.05. Similar observations were made by Damin et al. [35] who investigated the effect of cold storage (35 days) on various rheological properties of yoghurt such as firmness, G’ and G’’. Those authors observed increasing values for G’ and G’’ towards the end of the storage period. Serra et al. [34] also reported an increase in G’ when investigating the texture of set and stirred yoghurts during the first 14 days of storage. In the same context, Lucey et al. [37] hypothesized a presumably ongoing fusion of casein particles leading to an increase in viscoelastic parameters during storage.

### 3.4. Texture Profile Analysis by Sensory Evaluation

The heating of yoghurt gels after culturing was shown in the first part of this study to have just a minor effect on the aroma profile and no influence on the taste profile of the samples [12]. Since this second part of the study focuses on the effect of RF heating on the textural and microstructural properties of yoghurt, STPA (Sensory Texture Profile Analysis) was performed on the stirred samples as a complementary non-instrumental, human-sensory evaluation. The results are shown in Table 6. A trained sensory panel evaluated the creaminess, stickiness, homogeneity by mouthfeel and the visual homogeneity (“v”) over the entire storage period. For direct comparison of textural changes, a set-style reference yoghurt was also investigated (Week 0).

**Table 6:** Intensity rating of specific texture attributes by sensory texture profile analysis (STPA) of yoghurt gels with/without additional heat treatment after culturing and results of ANOVA (one-way): Weeks 0, 2, 4.

Sensory Texture Profile Analysis (STPA) (Visual Analogue Scale, Mean Values)		Reference Yoghurt	RF-Treated Yoghurts		CV-Treated Yoghurts		Set Reference	ANOVA Results	
			58 °C	65 °C	58 °C	65 °C		*F* Value	*p* Value
Week 0	Creaminess	6.6b	7.7ab	7.5ab	8.3a	7.7ab	4.8c	5.49	2.47 × 10^−4^
	Stickiness	7.6	7.5	7.1	6.8	6.5	6.9	0.41	8.42 × 10^−1^ *
	Homogeneity	8.8a	6.8b	5.3b	6.9b	5.9b	1.7c	14.80	5.69 × 10^−10^
	Homogeneity (v)	9.2a	8.3abc	7.3c	8.8ab	7.6bc	4.0d	16.30	1.00 × 10^−10^
Week 2	Creaminess	7.7ab	7.3abc	5.7c	8.2a	5.9bc	-	2.94	2.76 × 10^−2^
	Stickiness	7.7a	5.6b	4.8b	5.8ab	5.4b	-	2.43	5.76 × 10^−2^
	Homogeneity	8.2a	4.2bc	2.5c	5.2b	3.5bc	-	7.60	5.05 × 10^−5^
	Homogeneity (v)	9.5a	7.2b	3.8d	6.8bc	5.2cd	-	8.86	1.09 × 10^−5^
Week 4	Creaminess	8.1	7.0	8.2	7.4	6.5	-	1.43	0.24 *
	Stickiness	7.2	6.0	7.3	5.7	6.4	-	0.75	0.56 *
	Homogeneity	10.0a	7.1b	6.2bc	4.7cd	4.2d	-	15.31	3.07 × 10^−8^
	Homogeneity (v)	9.7a	8.5ab	8.5a	6.8bc	6.5c	-	5.23	1.34 × 10^−3^

* No *post-hoc* test necessary (*p* ≥ 0.10, n.s.). Intensity values with different letters indicate significant differences between products (*p* < 0.05, Fisher LSD *post-hoc*).

Besides the set-style reference, all the investigated yoghurt products had a creamy, smooth, and homogenous texture directly after manufacturing with only minor differences (Table 6). Regarding the creaminess, homogeneity, and visual homogeneity attributes, there was a significant difference between the set-style reference and the other samples, with the set-style reference being the least creamy and grainiest sample. These findings can be related to the different microstructure of set-style yoghurts [24]. A closer look at the STPA results of the heat-treated samples directly after manufacturing reveals only minor differences to the stirred reference with regard to the perceived creaminess. In detail, the heat-treated yoghurts were evaluated as being even slightly creamier than the reference (Table 6). In view of the visual homogeneity, only the yoghurts heated to 65 °C (RF, CV) were evaluated to be significantly different from the reference. In Section 3.1, the presence of white flakes/lumps was reported after post-fermentative heat treatment. In agreement with our STPA results, these flakes increased in number and size with increasing temperature and time of heat treatment.

A difference in the homogeneity of the overall mouthfeel was more pronounced after an additional heat treatment. These differences in the perceived homogeneity of the samples were presumably dependent on the microstructural changes of the samples described in Section 3.1: with increasing thermal stress, the structure of the network opened while instrumental texture analysis revealed increasing differences in the samples’ inner gel strength (Section 3.3: CO, % broken structure). As the perceived viscosity and consistency is reported to decrease with a loss of inner gel strength, this is also in good agreement with our results on homogeneity as rated by mouthfeel [38].

The stickiness attribute was rated quite differently. No significant differences between the heat-treated yoghurts and the stirred and set-style references were obtained. Moreover, even in storage the evaluation of this textural attribute remained very similar between all samples, with only small differences perceived by the panelists after two weeks of storage (Table 6). Likewise, the creaminess of all samples was also evaluated as being very similar between the samples after two weeks of storage, and no significant sensory differences of any kind were found after four weeks of storage.

Greater differences between the stirred reference and the other yoghurt samples were observed with storage time for the homogeneity and visual homogeneity attributes. However, this difference was not significant after four weeks of storage for the RF heat-treated yoghurts. It is noteworthy that the latter findings from the sensory texture evaluation agree quite well with the experimental results described in Section 3.3 where textural analysis confirmed that RF heated products were closer to the reference than were yoghurts after CV heating.

**Figure 5:**
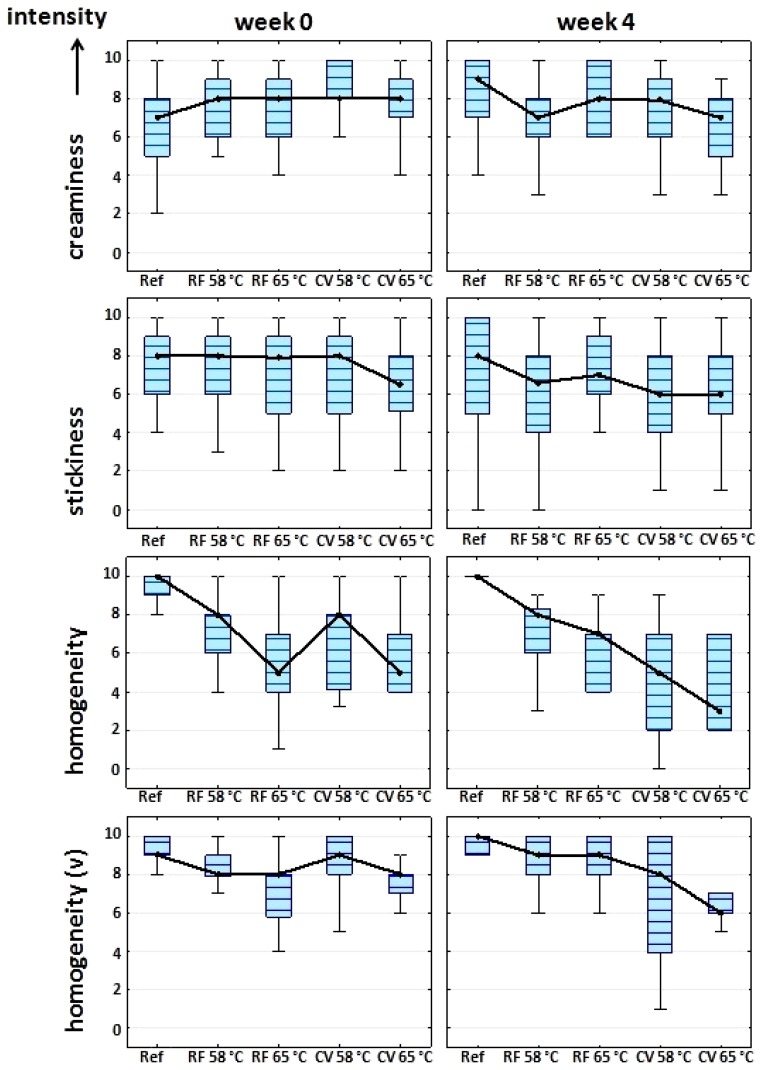
Box-plot of STPA data from a trained sensory panel, comparing different textural attributes of yoghurts directly after heat treatment and after four weeks’ storage. Median: •; whiskers: ±minimum-maximum ratings (without outliers); box: percentiles 25%–75%.

Figure 5 summarizes in more detail the results given in Table 6, comparing Week 0 and Week 4 and highlighting the panelists’ inter-individual variation. The data are shown as median values (instead of the arithmetic mean) in box-plot diagrams due to their robustness towards outliers. Figure 5 clearly shows that with regard to the homogeneity and visual homogeneity attributes the samples were evaluated as grainier by the panelists with increasing temperature and time of the heat treatment. These differences were even more pronounced after four weeks of storage and on highlighting the median values and the 25%–75% quartiles of the box-plots.

Differences in the homogeneity and visual homogeneity on longer storage were perceived by the panelists in the case of the CV heated yoghurts (Table 6, Figure 5). Panelists reported a tendency towards a grainier texture/mouthfeel; nevertheless, the ratings for the CV 58 °C yoghurt were not statistically significant (homogeneity: *F* = 2.26 (n.s.), 3.72 (sig.); homogeneity (visual): *F* = 3.23 (n.s.), 3.46 (sig.); *p* < 0.05 (CV 58 °C and CV 65 °C)).

### 3.5. Hedonic Rating of Stirred Yoghurt Gels

Besides the evaluation of the textural properties of the samples, the flavor and taste of the reference(s) and heat-treated samples were evaluated by a trained sensory panel according to the protocol previously described [12]. After completion of all the profile analyses, the panelists were additionally asked to rank the products’ popularity. The hedonic rating revealed the following ranking after four weeks of storage: reference (8, like very much) > RF 58 °C, RF 65 °C and CV 58 °C (7, like moderately) > CV 65 °C (6, like slightly). The highest discrepancies in popularity were observed between the reference product and the CV 65 °C product.

In the first part of our study [12], we also reported on a triangular testing that was additionally performed. The aim was to reveal if the differences in the yoghurts’ sensory profile ratings would make it more or less complicated for the panelists to distinguish the differently treated yoghurts. The testing was performed blindfolded so that visual properties such as differences in color and visual graininess did not influence the outcome of the triangle evaluation. The CV 65 °C yoghurt was excluded from the triangle test since its flavor and taste were found to differ significantly from the other samples and, as mentioned above, was the least liked in the hedonic ranking. When performing the triangular testing, it became evident that the panel could not perceive any statistically significant differences between the stirred reference sample and the RF and CV heated yoghurts with respect to the their overall sensory properties (flavor, taste, texture). Further details are described in our previous publication [12].

## 4. Conclusions

This second part of our study aimed to characterize potential microstructural and textural effects in stirred yoghurt caused by post-fermentative heat treatment to prolong yoghurts’ shelf-life, a procedure that is currently rarely used. In a comparative evaluation, the focus was on the effects of CV and RF heating. Microstructural changes, such as changes in pore radii and increases in the free serum phase were observed which increased with increasing thermal stress. Syneresis, textural changes, and popularity of the products were shown to be in agreement with cryo-SEM images: RF treatment produced yoghurts were more similar to the stirred reference product than after CV heat treatment. One major drawback of even gentle additional heat treatment by RF was found to be the formation of white flakes and a decrease in the inner gel strength. Nevertheless, textural defects such as white flakes are known to be reversible. A further improvement could possibly be achieved by an additional homogenization step after heat treatment, for example by pumping the curd through continuous flow coolers or special devices.

Overall, this two-part feasibility study was able to characterize the microbial, sensorial, textural, and microstructural changes caused by post-fermentative heat treatment, and such data has not hitherto been reported in the literature. A quick, homogeneous, and gentle RF treatment up to 65 °C was shown to prolong the shelf-life of yoghurts by reduced microbial numbers while only minor sensorial and presumably reversible textural changes were observed [12]. Such findings on less susceptible yoghurt products with satisfactory sensorial and textural properties open up opportunities for improved “heat treated” yoghurt sales, especially in countries where the distribution cold chain cannot be guaranteed at all times. In a follow-up step, radio frequencies could be applied to stirred yoghurt in an RF tube. The application of radio frequencies in a continuous manufacturing process would open up this technique to industry, while the yoghurts’ textural and sensorial properties would be expected to be further improved.

## Abbreviations

A: area under curve
AD: adhesiveness (g·s)
ANOVA: analysis of variance
CO: cohesiveness
cryo-SEM: cryo-scanning electron microscopy
CV: convectional heating
F: force (g)
GU: gumminess (g)
HD: hardness (g)
ND: not detected
n.s.: not significant
RF: radio frequency heating
sig.: significant
STPA: sensory texture profile analysis
TPA: texture profile analysis
